# Ipragliflozin Improves Hepatic Steatosis in Obese Mice and Liver Dysfunction in Type 2 Diabetic Patients Irrespective of Body Weight Reduction

**DOI:** 10.1371/journal.pone.0151511

**Published:** 2016-03-15

**Authors:** Chikara Komiya, Kyoichiro Tsuchiya, Kumiko Shiba, Yasutaka Miyachi, Shunsaku Furuke, Noriko Shimazu, Shinobu Yamaguchi, Kazuo Kanno, Yoshihiro Ogawa

**Affiliations:** 1 Department of Molecular Endocrinology and Metabolism, Graduate School of Medical and Dental Sciences, Tokyo Medical and Dental University, Tokyo, Japan; 2 Kanno Clinic, Tokyo, Japan; 3 Japan Agency for Medical Research and Development, CREST, Tokyo, Japan; Cincinnati Children's Hospital Medical Center, UNITED STATES

## Abstract

Type 2 diabetes mellitus (T2DM) is associated with a high incidence of non-alcoholic fatty liver disease (NAFLD) related to obesity and insulin resistance. Currently, medical interventions for NAFLD have focused on diet control and exercise to reduce body weight, and there is a requirement for effective pharmacological therapies. Sodium-glucose cotransporter 2 (SGLT2) inhibitors are oral antidiabetic drugs that promote the urinary excretion of glucose by blocking its reabsorption in renal proximal tubules. SGLT2 inhibitors lower blood glucose independent of insulin action and are expected to reduce body weight because of urinary calorie loss. Here we show that an SGLT2 inhibitor ipragliflozin improves hepatic steatosis in high-fat diet-induced and leptin-deficient (*ob/ob*) obese mice irrespective of body weight reduction. In the obese mice, ipragliflozin-induced hyperphagia occurred to increase energy intake, attenuating body weight reduction with increased epididymal fat mass. There is an inverse correlation between weights of liver and epididymal fat in ipragliflozin-treated obese mice, suggesting that ipragliflozin treatment promotes normotopic fat accumulation in the epididymal fat and prevents ectopic fat accumulation in the liver. Despite increased adiposity, ipragliflozin ameliorates obesity-associated inflammation and insulin resistance in epididymal fat. Clinically, ipragliflozin improves liver dysfunction in patients with T2DM irrespective of body weight reduction. These findings provide new insight into the effects of SGLT2 inhibitors on energy homeostasis and fat accumulation and indicate their potential therapeutic efficacy in T2DM-associated hepatic steatosis.

## Introduction

Type 2 diabetes mellitus (T2DM) is an epidemic associated with an increasing prevalence of obesity [1–3]. Whereas patients with T2DM are at a high risk of vascular diseases [4], non-vascular diseases such as certain cancers and infectious diseases are often associated with T2DM. Liver diseases, including liver cirrhosis and hepatocellular carcinoma, are one of the leading causes of death in addition to cardiovascular diseases in T2DM patients [5]. Non-alcoholic fatty liver disease (NAFLD) is understood as excessive lipid accumulation in the liver associated with obesity and insulin resistance, and it is the most common chronic liver disease worldwide [6]. NAFLD includes a spectrum ranging from simple steatosis to non-alcoholic steatohepatitis (NASH), a severe form of NAFLD histologically characterized by steatosis with lobular inflammation and fibrosis. Approximately 10%–20% of patients with NAFLD develop NASH, and it can progress to cirrhosis and hepatocellular carcinoma. In patients with T2DM, the prevalence of NAFLD is as high as 75%, and they are prone to develop NASH [7]. However, no established pharmacological treatments are currently used for NAFLD, and medical interventions have focused on diet control and exercise [8]. Therefore, there is a requirement for effective pharmacological therapies.

Sodium-glucose cotransporter 2 (SGLT2) inhibitors are newly-developed oral antidiabetic drugs. SGLT2 is primarily expressed in renal proximal tubules, where it reabsorbs approximately 90% of glucose filtered at the renal glomeruli. SGLT2 inhibitors lower blood glucose independent of insulin action by facilitating the excretion of glucose in urine, and these inhibitors are expected to cause a reduction in body weight because of urinary calorie loss [9–11]. In terms of a role in improving obesity, SGLT2 inhibitors could be useful for the treatment of NAFLD. However, a recent study has reported that the SGLT2 inhibitor empagliflozin enhanced appetite and energy intake in patients with T2DM, attenuating the effect on body weight reduction [12]. An animal study has shown that diet-induced obese rats treated with the SGLT2 inhibitor dapagliflozin did not show a decrease in their body weight as predicted because of hyperphagia [13]. Although previous reports have demonstrated that the SGLT2 inhibitors ipraglifozin [14] and luseogliflozin [15] improved diet-induced hepatic steatosis in a mouse model with impaired insulin secretion, it is unknown whether SGLT2 inhibitors improve hepatic steatosis in an obese diabetic mouse model with insulin resistance, and if so, whether it depends on body weight reduction. Furthermore, there have been no clinical studies to examine the association between the changes of liver dysfunction and body weight in T2DM patients treated with SGLT2 inhibitors.

During SGLT2 inhibitor treatment, alteration of metabolism in various organs of the body is anticipated in response to urinary glucose excretion. In obesity, excess energy accumulates as triglycerides not only in adipose tissue but also ectopically in non-adipose tissues; especially, ectopic lipid accumulation in liver has strongly been associated with whole-body and tissue-specific insulin resistance and inflammatory processes [16]. However, it is completely unknown which lipid, in adipose tissue or liver, are more susceptible to the effect of urinary calorie loss induced by SGLT2 inhibitors. In addition, it is unclear where increased energy intake by hyperphagia is accumulated with SGLT2 inhibitor treatment, and how the lipid accumulation affects systemic and tissue glucose metabolism.

In this study, we show that ipragliflozin improved hepatic steatosis in diet-induced and genetically leptin-deficient (*ob/ob*) obese mice irrespective of body weight reduction. Ipragliflozin induced hyperphagia to attenuate body weight reduction in these mice, associated with increased epididymal fat mass. Despite increased adiposity, ipragliflozin ameliorated obesity-associated inflammation and insulin resistance in the epididymal fat. Clinically, ipragliflozin treatment improved liver dysfunction in patients with T2DM irrespective of body weight reduction. Our data provide evidence for the novel actions of SGLT2 inhibitors on energy homeostasis and fat accumulation in obesity and suggest their potential therapeutic efficacy in T2DM-associated hepatic steatosis.

## Materials and Methods

### Ethics Statement

This study was carried out in strict accordance with the guidelines for the care and use of laboratory animals of Tokyo Medical and Dental University. The protocol was approved by Tokyo Medical and Dental University Committee on Animal Research (No. 2013-025C4, No. 0160068A). The clinical study protocol was approved by the ethical committee on Kanno Clinic and Tokyo Medical and Dental University (No. 2204). All participants provided written informed consent, and all samples and clinical data were collected for clinical practice at Kanno Clinic. We disclosed detailed information on the study protocol, and provided all participants with an opportunity to refuse their inclusion in the study.

### Animals and experimental protocol

Male C57BL/6J wild-type (WT) mice were purchased from CLEA Japan, Inc. (Tokyo, Japan), Charles River Laboratories Japan, Inc. (Kanagawa, Japan), and Japan SLC, Inc. (Shizuoka, Japan). Male C57BL/6J-*ob/ob* (*ob/ob)* mice were purchased from Japan SLC, Inc. The animals were allowed free access to water and a standard diet (SD, CE-2; 343 kcal/100g, 12.6% energy as fat; CLEA Japan, Inc.). Ipragliflozin (provided by Astellas Pharma Inc., Tokyo, Japan) was dissolved in dimethyl sulfoxide (DMSO; Nacalai Tesque, Inc., Kyoto, Japan) and added into the drinking water. In the high-fat diet (HFD) feeding experiments, 8-week-old WT mice were fed a HFD (D12492; 524 kcal/100g, 60% energy as fat; Research Diets, Inc., New Brunswick, NJ, USA) for 12 weeks, and thereafter a HFD with the vehicle or ipragliflozin for 4 weeks. A pair-feeding experiment was performed by measuring the food intake of vehicle-treated mice fed a HFD *ad libitum* every 24 h (just after onset of the light cycle daily). The following day, the ipragliflozin-treated mice pair-fed a HFD were given the average amount of food consumed by the vehicle-treated mice fed a HFD *ad libitum* on the previous day. In a study using *ob/ob* mice, 7-week-old *ob/ob* mice fed a SD received the vehicle or ipragliflozin for 4 weeks. Age-matched control WT mice were fed a SD throughout the experiment period. Body weight, food and water intake, and blood glucose were measured every week. The dose of ipragliflozin was estimated based on daily water consumption and body weight. Concentration of ipragliflozin in the drinking water was changed every week to adjust 10 mg/kg/day; the average amount of ipragliflozin consumed during the study was 11 mg/kg/day. At the end of the experiment, the animals were sacrificed under intraperitoneal pentobarbital anesthesia (30 mg/kg) after 16 h of fasting.

### Biochemical assays

Blood glucose was measured using a glucometer (Glutest PRO R; Sanwa Kagaku Kenkyusho Co., Ltd., Aichi, Japan). Serum non-esterified fatty acid (NEFA), triglyceride (TG), and 3-hydroxybutyrate levels were determined with NEFA C-Test Wako (Wako Pure Chemical Industries, Ltd., Osaka, Japan), TG E-Test Wako (Wako Pure Chemical Industries, Ltd.), and beta Hydroxybutyrate Assay Kit (Abcam plc, Cambridge, UK), respectively. Serum alanine aminotransferase (ALT) levels were measured using Fuji Dry-chem 7000V (Fujifilm corporation, Tokyo, Japan). Urine glucose levels were analyzed with enzymatic assays in a laboratory of Oriental Yeast Co., Ltd. (Tokyo, Japan). Serum insulin and plasma glucagon levels were measured with an enzyme-linked immunosorbent assay kit (Morinaga Institute of Biological Science, Inc., Kanagawa, Japan) and Mercodia Glucagon ELISA (Mercodia AB, Uppsala, Sweden), respectively. Total lipids were extracted from the liver with chloroform and methanol (2:1 v/v), and liver TG content was assayed with TG E-Test Wako.

### Histological analysis

The liver and epididymal fat were fixed with 4% paraformaldehyde and embedded in paraffin. Liver sections were stained with hematoxylin and eosin (HE). For the measurement of adipocyte cell size, > 250 cells were counted per each section using an image analysis software (WinROOF; Mitani Corporation, Tokyo, Japan). Macrophages in the epididymal fat were immunohistochemically detected using a rat monoclonal F4/80 antibody (MCA497GA; Abd Serotec, Kidlington, UK). The density of crown-like structures (CLS) was obtained by counting the total numbers of CLS and adipocytes in each section and expressed as percentage [17, 18].

### Quantitative RT-PCR

Total RNA of the liver and epididymal fat was isolated using Sepasol reagent (Nacalai Tesque, Inc.) and RNeasy Plus Universal Mini Kit (Qiagen, Hilden, Germany), respectively. RNA was reverse transcribed with Random Primer (Thermo Fisher Scientific Inc., Waltham, MA, USA) and ReverTra Ace (Toyobo Co., Ltd., Osaka, Japan). Quantitative RT-PCR was performed using StepOnePlus Real-time PCR System with Fast SYBR Green Master Mix Reagent (Thermo Fisher Scientific Inc.). Primers are listed in S1 Table. Data were normalized to the *36b4* levels, and analyzed by the comparative CT method.

### Analysis of insulin signaling

Mice were injected with 5 U/kg of human insulin (Humulin R, Eli Lilly and Company, Indianapolis, IN, USA) via the portal vein after 16 h of fasting. The liver, epididymal fat, and skeletal muscle were homogenized in a lysis buffer (2% SDS, 4 M Urea, 1 mM EDTA, 150 mM NaCl, 50 mM Tris pH 8.0) supplemented with Halt Protease and Phosphatase Inhibitor Cocktail (Thermo Fisher Scientific Inc.). Immunoblotting was performed with a phospho (Ser473)-Akt (9271, Cell Signaling Technology, Danvers, MA, USA) and a total Akt antibody (9272, Cell Signaling Technology). Immunoblots were detected and analyzed with ECL Prime Western Blotting Detection Reagent and ImageQuant LAS 4000 mini (GE Healthcare, Little Chalfont, UK).

### Clinical study

From June 2014 to March 2015, 55 patients with T2DM, age > 20 years, with body mass index (BMI) > 22 kg/m^2^ and hemoglobin A_1c_ (HbA_1c_) > 6.5% were enrolled at the diabetes clinic (Kanno Clinic, Tokyo, Japan). Twenty-five of 55 patients were diagnosed as hepatic steatosis by ultrasonography before the study. Exclusion criteria were SGLT2 inhibitors treatment history, urinary tract infection, and pituitary or adrenal insufficiency. Age, sex, height, body weight, BMI, waist circumference, blood pressure, duration of diabetes, medications, and comorbidities were recorded at the beginning of the study (baseline). After 24 weeks of ipragliflozin (50 mg/day) treatment, we divided the patients into tertiles according to percentage of body weight change from baseline. Tertile 1 was defined as those with the highest tertile of body weight reduction, Tertile 2 as the intermediate, and Tertile 3 as the lowest. Fasting plasma glucose (FPG), HbA_1c_, serum levels of ALT, γ-GTP, TG, and high- and low-density lipoprotein (HDL and LDL, respectively) cholesterol were measured in a clinical laboratory of Syowa Medical Science Corporation (Tokyo, Japan) at baseline and 12 and 24 weeks after treatment. The anonymized and de-identified clinical dataset is available upon request.

### Statistical analysis

Data are expressed as mean ± standard error of the mean (SEM). Data were compared using chi-square test, student’s *t* test, or analysis of variance (ANOVA) with post hoc testing. Pearson correlation coefficient was used to evaluate correlations between variables. *p* < 0.05 was considered to be statistically significant. Statistical analysis was performed using Prism 6 (GraphPad software, Inc., La Jolla, CA, USA).

## Results

### Ipragliflozin improves hepatic steatosis irrespective of body weight reduction in obese mice with insulin resistance

**Fig 1:**
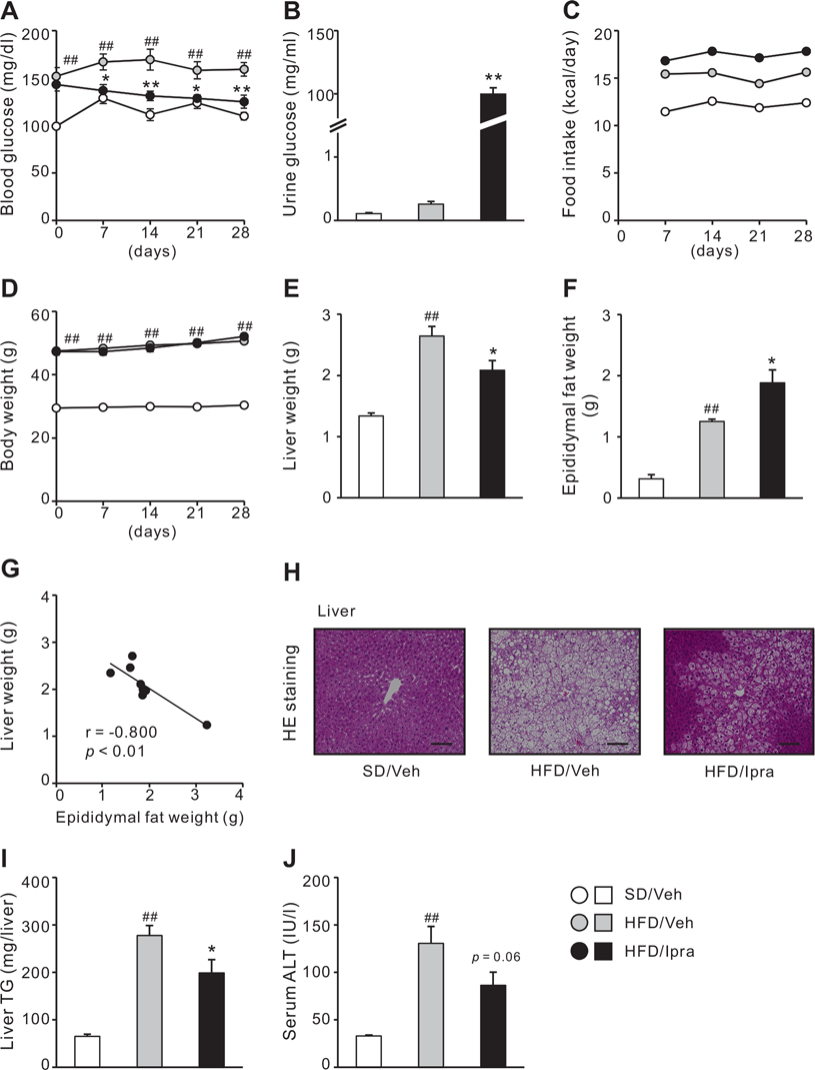
Metabolic characterization and hepatic steatosis of HFD-fed WT mice treated with or without ipragliflozin. Eight-week-old WT mice were fed a SD or a HFD for 16 weeks. Mice were given with the vehicle or 10mg/kg of ipragliflozin during last 4 weeks. (A) Blood glucose during ipragliflozin treatment. (B) Urine glucose concentration after 3 weeks of ipragliflozin treatment. (C) Food intake and (D) body weight during ipragliflozin treatment. Weights of the (E) liver and (F) epididymal fat after 4 weeks of ipragliflozin treatment. (G) Correlation of the liver weight with the epididymal fat weight in ipragliflozin-treated mice. (H) Hematoxylin and eosin (HE) staining and (I) triglyceride (TG) content of the liver. (J) Serum ALT levels. Veh, vehicle; Ipra, ipragliflozin. Original magnification, × 200. Scale bars, 100 μm. # *p* < 0.05, ## *p* < 0.01 vs SD/Veh; * *p* < 0.05, ** *p* < 0.01 vs HFD/Veh. *n* = 6–8.

To examine the effects of ipragliflozin on insulin resistance- and obesity-associated metabolic phenotypes, ipragliflozin or the vehicle were orally administered for 4 weeks to WT mice fed a HFD. Ipragliflozin consistently improved HFD-induced hyperglycemia during the treatment, in conjunction with an increase in urinary glucose excretion (Fig 1A and 1B, Table 1) and water intake (data not shown). Ipragliflozin increased energy intake with hyperphagia, and the changes in body weight were consequently comparable between ipragliflozin- and vehicle-treated mice (Fig 1C and 1D). After 4 weeks of treatment, fasting hyperglycemia and hyperinsulinemia in HFD-fed mice were significantly improved, and fasting serum NEFA, 3-hydroxybutyrate, and plasma glucagon levels were significantly increased by ipragliflozin treatment (Table 1). Whereas liver weight was significantly decreased by ipragliflozin compared with the vehicle, epididymal fat weight was increased (Fig 1E and 1F). There was a significant negative correlation between liver and epididymal fat weights in ipragliflozin-treated mice (Fig 1G). Consistent with the decreased liver weight, hepatic lipid deposition and TG content were significantly attenuated in ipragliflozin-treated mice (Fig 1H and 1I). Furthermore, ipragliflozin-treated mice showed a decreasing trend in serum ALT levels (Fig 1J). Similar results were obtained in *ob/ob* mice (S1 Fig).

**Table 1:** Metabolic parameters of SD- or HFD-fed WT mice treated with or without ipragliflozin for 4 weeks.

	SD/Veh		HFD/Veh		HFD/Ipra	
	Fed	Fasted	Fed	Fasted	Fed	Fasted
Blood glucose (mg/dl)	149.9 ± 4.7	72.2 ± 2.2	160.1 ± 6.3	128.1 ± 6.6 ##	145.4 ± 5.2	78.9 ± 3.9 **
Serum NEFA (mEq/l)	0.85 ± 0.09	2.12 ± 0.07	1.12 ± 0.09	1.29 ± 0.05 ##	1.04 ± 0.07	1.51 ± 0.07 *
Serum TG (mg/dl)	80.3 ± 8.8	69.0 ± 4.4	83.1 ± 4.5	58.0 ± 2.9	103.9 ± 8.3	64.5 ± 7.4
Serum 3-hydroxybutyrate (mmol/l)	0.48 ± 0.03	0.55 ± 0.04	0.65 ± 0.08	1.64 ± 0.12 ##	1.69 ± 0.14	2.42 ± 0.09 **
Serum insulin (ng/ml)	0.60 ± 0.14	0.25 ± 0.03	20.01 ± 4.73 ##	2.09 ± 0.12 ##	6.23 ± 1.58 *	0.93 ± 0.12 **
Plasma glucagon (pg/ml)		6.31 ± 1.64		5.25 ± 0.82		13.84 ± 2.65 **

SD, standard diet; HFD, high-fat diet; Veh, vehicle; Ipra, ipragliflozin; NEFA, non-esterified fatty acid; TG, triglyceride. Data are mean ± SEM.

## *p* < 0.01 vs SD/Veh

* *p* < 0.05

** *p* < 0.01 vs HFD/Veh. *n* = 6–8.

To examine the effect of ipragliflozin-induced hyperphagia on metabolic phenotypes, we conducted a pair-feeding experiment. The pair-feeding experiment revealed that ipragliflozin-treated mice pair-fed a HFD showed significant weight loss as well as improved hyperglycemia compared with vehicle-treated mice fed a HFD *ad libitum* (S2A and S2B Fig). The observation suggests that ipragliflozin-induced increase of energy intake offset urinary calorie loss, resulting in comparable body weight between ipragliflozin- and vehicle-treated mice fed a HFD. Ipragliflozin-treated mice pair-fed a HFD displayed significant decrease of liver weight, and non-significant increase of epididymal fat weight (S2C and S2D Fig). Ipragliflozin attenuated hepatic lipid deposition and TG content in pair-fed mice fed a HFD compared with vehicle-treated mice fed a HFD *ad libitum* (S2E and S2F Fig). Ipragliflozin reduced fasting blood glucose and serum insulin levels in pair-fed mice fed a HFD compared with vehicle-treated mice fed a HFD *ad libitum* (S2G and S2H Fig). Plasma glucagon levels in ipragliflozin-treated mice pair-fed a HFD were higher than those in vehicle-treated mice fed a HFD *ad libitum*, but it did not reach statistical significance (S2I Fig).

These observations indicate that ipragliflozin improves glucose intolerance and hepatic steatosis irrespective of body weight reduction in obese mice with insulin resistance. Ipragliflozin enhanced appetite and energy intake in the obese mice to attenuate body weight reduction by urinary calorie loss, associated with increased epididymal fat mass.

### Ipragliflozin inhibits lipogenic and macrophage marker gene induction in the liver of HFD-fed mice

**Fig 2:**
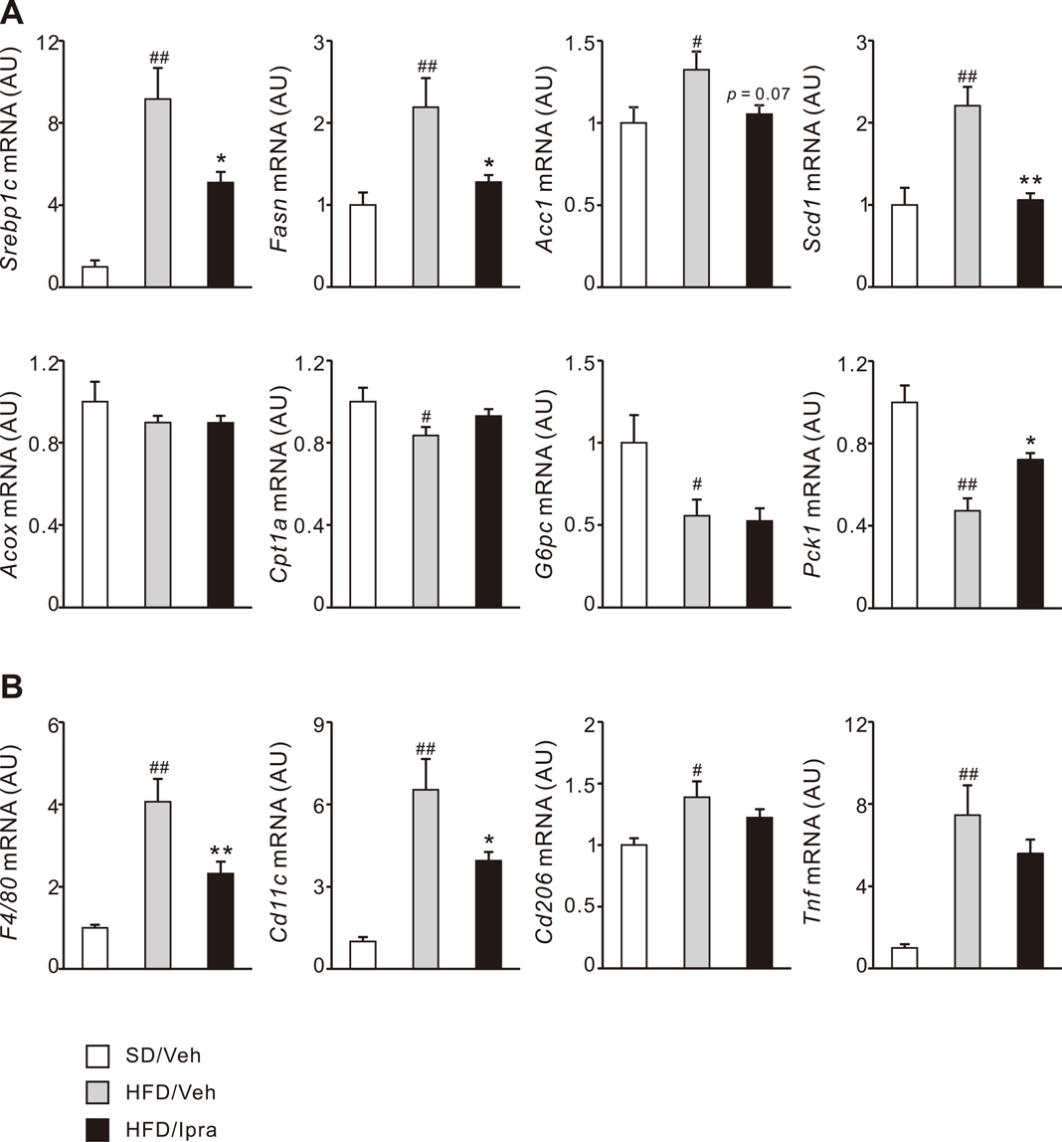
Effect of ipragliflozin on liver gene expression in HFD-fed WT mice. Gene expression levels in the liver associated with (A) lipogenesis, β-oxidation, gluconeogenesis, and (B) inflammation after 4 weeks of the vehicle or ipragliflozin treatment. # *p* < 0.05, ## *p* < 0.01 vs SD/Veh; * *p* < 0.05, ** *p* < 0.01 vs HFD/Veh. *n* = 6–8.

To elucidate the mechanisms by which ipragliflozin strongly prevented hepatic steatosis in the obese mice, we examined gene expressions involved in fatty acid and glucose metabolism in the liver. Expressions of *de novo* lipogenic genes such as sterol regulatory element-binding protein-1c (*Srebp1c*), fatty acid synthase (*Fasn*), acetyl-CoA carboxylase 1 (*Acc1*), and stearoyl-CoA desaturase 1 (*Scd1*) were upregulated in HFD-fed mice compared with SD-fed mice, which was significantly suppressed by ipragliflozin treatment (Fig 2A). Expression of β-oxidation-related genes such as acyl-CoA oxidase 1, palmitoyl (*Acox1*) and carnitine palmitoyltransferase 1A (*Cpt1a*) were not affected by ipragliflozin treatment in HFD-fed mice (Fig 2A). Ipragliflozin treatment upregulated expression of the gluconeogenic gene, phosphoenolpyruvate carboxykinase 1 (*Pck1*), in HFD-fed mice (Fig 2A). Obesity-induced hepatic inflammation was demonstrated by elevated gene expression of the common macrophage marker *F4/80*, which was inhibited by ipragliflozin treatment (Fig 2B). Ipragliflozin treatment downregulated gene expression of the pro-inflammatory macrophage M1 marker *Cd11c* in HFD-fed mice, without affecting that of the anti-inflammatory macrophage M2 marker *Cd206* (Fig 2B). Gene expression of tumor necrosis factor-α (*Tnf*) also showed a decreasing tendency in ipragliflozin-treated mice compared with vehicle-treated mice (Fig 2B). These data suggest that ipragliflozin prevents obesity-associated hepatic steatosis and inflammation with decreased *de novo* lipogenesis and macrophage accumulation, respectively.

### Ipragliflozin attenuates adipose tissue inflammation in HFD-fed mice

**Fig 3:**
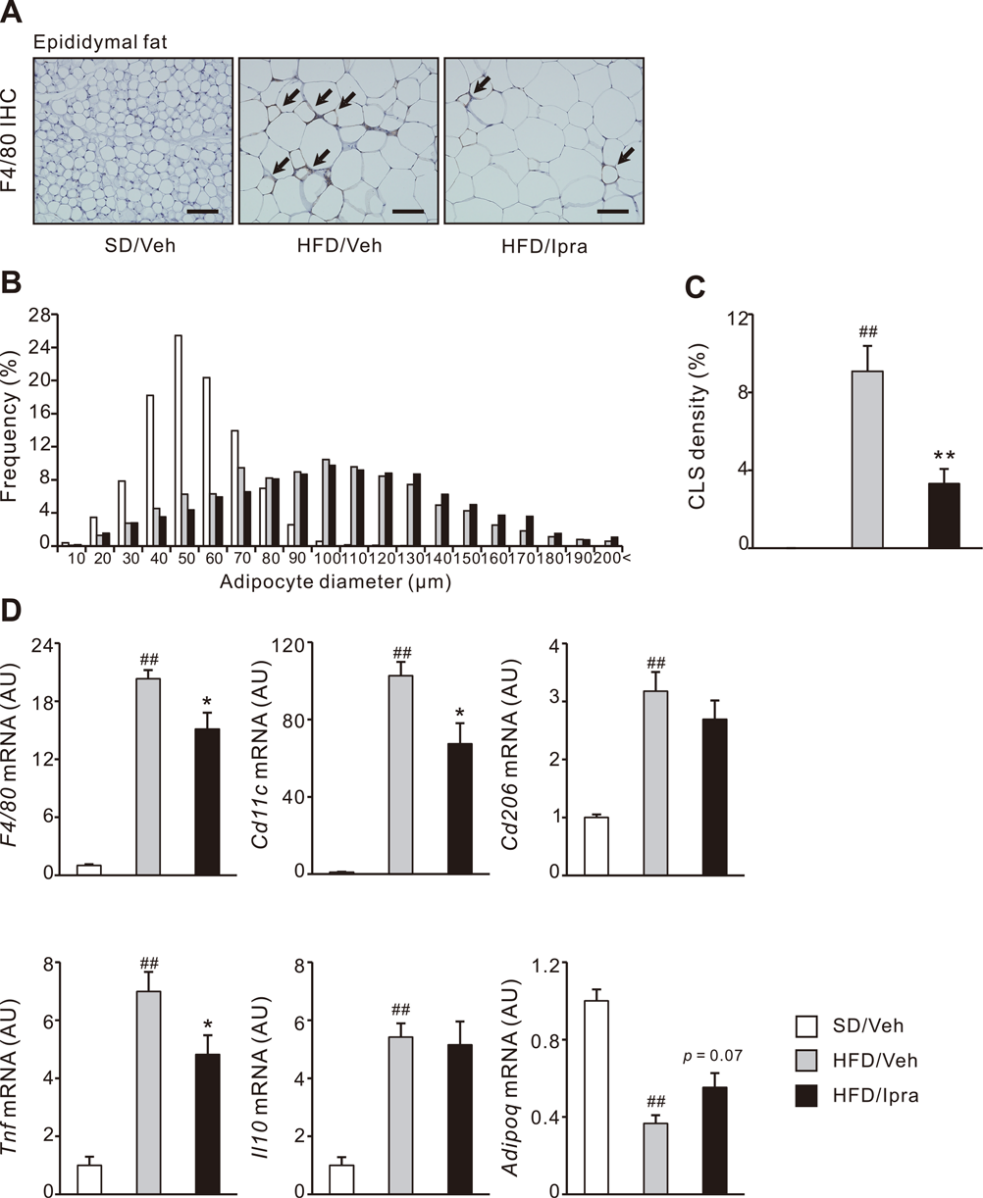
Effect of ipragliflozin on adipose tissue inflammation in HFD-fed WT mice. (A) F4/80 immunohistochemistry of the epididymal fat sections after 4 weeks of the vehicle or ipragliflozin treatment. Arrows indicate CLS. (B) Histogram of adipocyte diameter and (C) quantification of CLS density in the epididymal fat. (D) Gene expression levels in the epididymal fat associated with inflammation and adipocytokines. Original magnification, × 200. Scale bars, 100 μm. # *p* < 0.05, ## *p* < 0.01 vs SD/Veh; * *p* < 0.05, ** *p* < 0.01 vs HFD/Veh. *n* = 6–8.

Consistent with increased epidydimal fat mass in ipragliflozin-treated mice, histological analysis revealed that ipragliflozin treatment promoted adipocyte hypertrophy assessed by cell size in HFD-fed mice (Fig 3A and 3B). Despite adipocyte hypertrophy, the number of crown-like structures (CLS), which is a histological feature of a dead adipocyte surrounded with macrophages [19], was markedly decreased with ipragliflozin treatment in HFD-fed mice (Fig 3C). Gene expression of *F4/80*, *Cd11c*, and *Tnf* were upregulated in the epididymal fat of HFD-fed mice compared with SD-fed mice, which were inhibited by ipragliflozin treatment (Fig 3D). Adiponectin (*Adipoq*) gene expression showed an increasing trend in ipragliflozin-treated mice compared with vehicle-treated mice (Fig 3D). Ipragliflozin treatment did not alter gene expression of *Cd206* and *Il10* in the epididymal fat of HFD-fed mice (Fig 3D). These data indicate that increased epididymal fat mass does not deteriorate macrophage accumulation nor inflammation in adipose tissue of ipragliflozin-treated mice.

### Ipragliflozin improves insulin resistance in the liver and adipose tissue of HFD-fed mice

**Fig 4:**
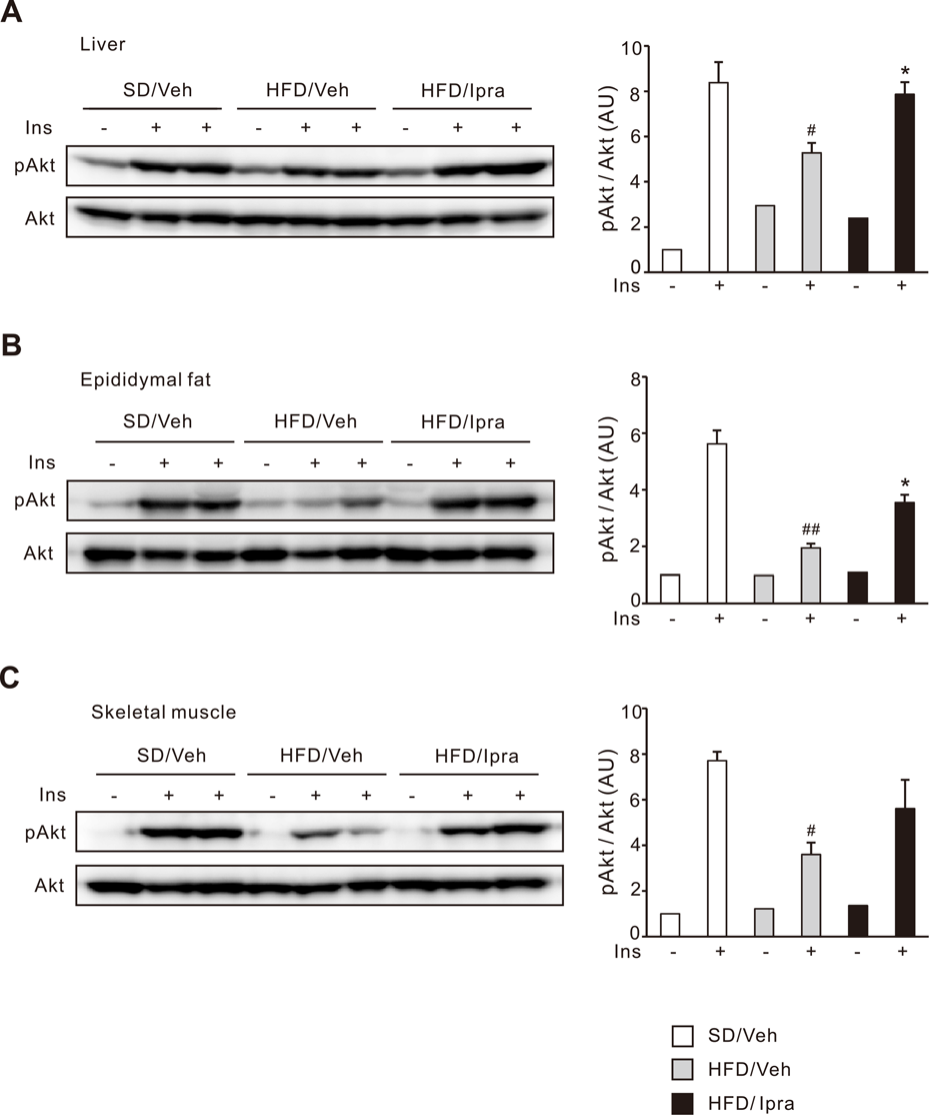
Effect of ipragliflozin on insulin signaling in HFD-fed WT mice. Representative immunoblots and quantification of phosphorylated Akt (S473) in the (A) liver, (B) epididymal fat, and (C) skeletal muscle after 4 weeks of the vehicle or ipragliflozin treatment. After 16 h of fasting, mice were injected with insulin (5U/kg) or phosphate buffered saline to portal vein, and the liver was collected 3 minutes later. Ins, insulin. # *p* < 0.05, ## *p* < 0.01 vs SD/Veh; * *p* < 0.05 vs HFD/Veh. *n* = 2–6.

We further examined whether altered lipid accumulation in the liver and adipose tissue of ipragliflozin-treated mice affected insulin sensitivity in these tissues. Insulin-induced phosphorylation of Akt was significantly increased in the liver of ipragliflozin-treated mice compared with vehicle-treated mice (Fig 4A). Phosphorylated Akt levels was also significantly improved in the epididymal fat of ipragliflozin-treated mice (Fig 4B). In the skeletal muscle, phosphorylated Akt levels tended to increase in ipragliflozin-treated mice, which did not reach statistical significance (Fig 4C). Possibly reflecting improved hyperinsulinemia, basal phosphorylated Akt levels in the liver of ipragliflozin-treated mice pair-fed a HFD are lower than those of vehicle-treated mice fed a HFD *ad libitum* (S3 Fig). These data indicate that ipragliflozin treatment attenuates hepatic steatosis in HFD-fed mice along with improved insulin resistance. In addition, despite increased fat mass and adipocyte size, ipragliflozin treatment also improved insulin resistance in the epididymal fat of HFD-fed mice.

### Ipragliflozin improves liver dysfunction irrespective of body weight reduction in patients with T2DM

**Fig 5:**
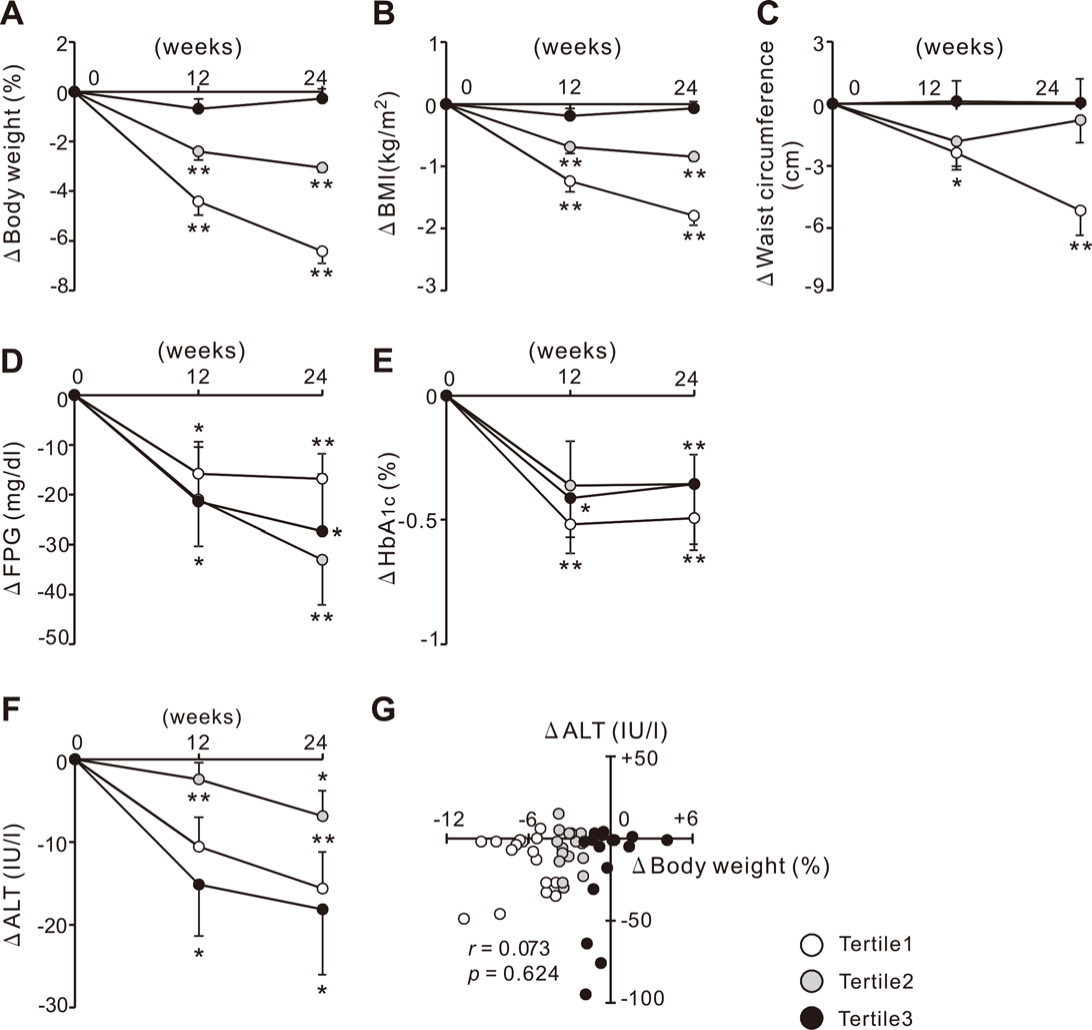
Changes in metabolic parameters and serum ALT levels in patients with T2DM during 24 weeks of ipragliflozin treatment. Changes in (A) body weight, (B) BMI, (C) waist circumference, and levels of (D) FPG, (E) HbA_1c_, and (F) serum ALT in patients with T2DM during 24 weeks of ipragliflozin treatment. (G) Correlation between changes in serum ALT levels and body weight after 24 weeks of ipragliflozin treatment. * *p* < 0.05, ** *p* < 0.01 vs baseline.

Based on the observations of the animal study, we conducted a clinical study to examine whether ipragliflozin improved T2DM-associated liver dysfunction irrespective of body weight reduction. Seven out of 55 patients (13%) were excluded because of adverse events (*n* = 3, 5%), non-compliance (*n* = 3, 5%), and loss to follow up (*n* = 1, 2%). Baseline characteristics of 48 patients (Total) and stratified groups (Tertiles 1–3) are shown in Table 2. Ninety-four percent of patients were already receiving antidiabetic medications, including metformin, thiazolidinediones, sulfonylureas, dipeptidyl peptidase-4 (DPP-4) inhibitors, α-glucosidase inhibitors, and insulins. One patient was receiving pioglitazone, and no one received glucagon-like peptide-1 (GLP-1) analogues. At baseline, although HbA_1c_ in Tertile 1 was lower than Tertile 2 and 3, age, duration of T2DM, medications, body weight, BMI, waist circumference, FPG, ALT, γ-GTP, and lipid profiles were comparable among all tertiles (Table 2). At week 24, body weight, BMI, and waist circumference were significantly reduced in Total, Tertile 1, and Tertile 2 compared with the baseline, but not in Tertile 3 (Figs 5A–5C, S4A–S4D and S6A). FPG was improved in Total and all tertiles (Figs 5D and S5A), and HbA_1c_ was also improved in Total, Tertile 1, and Tertile 2, and the trend was observed in Tertile 3 without statistical significance (Figs 5E and S5B). Consistent with the animal study, serum ALT levels were significantly decreased in Tertile 3, as well as Total, Tertile1, and Tertile 2 (Figs 5F and S5F). In addition, serum γ-GTP levels were significantly decreased in Total and Tertile 1, and a decreasing trend was observed in Tertile 3 (S5G and S6G Figs). The change in serum ALT levels did not correlate with the change in body weight in Total (Fig 5G) nor each tertile (data not shown). A subgroup analysis of the 25 patients (S2 Table), who were diagnosed as hepatic steatosis by ultrasonography before the study, confirmed that treatment of ipragliflozin also significantly improved liver dysfunction assessed by serum ALT levels (S7A Fig). The change in serum ALT levels did not correlate with the change in body weight in the subgroup, suggesting that the improvement of liver dysfunction is irrespective of body weight reduction in the subgroup (S7B Fig). Collectively, these clinical data indicate that ipragliflozin improves liver dysfunction irrespective of the change in body weight in patients with T2DM.

**Table 2:** Baseline characteristics of the patients with T2DM.

	Total	Tertile 1	Tertile 2	Tertile 3	*p* value
*n* (Male/Female)	48 (23/25)	16 (7/9)	16 (8/8)	16 (8/8)	0.920
Age (year)	54.3 ± 1.9	57.4 ± 3.8	52.5 ± 2.4	53.0 ± 3.6	0.528
Duration of diabetes (year)	10.8 ± 1.4	9.9 ± 2.4	9.5 ± 1.4	13.0 ± 3.3	0.553
Body weight (kg)	75.4 ± 2.1	76.2 ± 4.2	75.6 ± 4.6	74.4 ± 1.6	0.947
BMI (kg/m^2^)	28.5 ± 0.6	28.2 ± 1.1	28.7 ± 1.4	28.6 ± 0.8	0.940
Waist circumference (cm)	97.6 ± 1.4	99.3 ± 2.6	96.3 ± 2.9	97.3 ± 1.8	0.681
SBP (mmHg)	137.3 ± 2.2	133.5 ± 3.5	136.4 ± 3.7	141.9 ± 4.0	0.281
DBP (mmHg)	81.2 ± 1.4	77.8 ± 2.3	80.8 ± 2.5	84.9 ± 2.2	0.101
FPG (mg/dl)	151.3 ± 6.1	134.9 ± 7.7	157.4 ± 12.1	161.5 ± 11.1	0.166
HbA_1c_ (%)	7.73 ± 0.15	7.19 ± 0.16	8.00 ± 0.29	8.01 ± 0.27	0.034
ALT (IU/l)	41.8 ± 4.5	38.9 ± 6.9	34.9 ± 5.5	51.6 ± 9.9	0.288
γ-GTP (IU/l)	48.2 ± 5.9	42.8 ± 11.8	42.9 ± 7.8	59.2 ± 10.6	0.445
HDL cholesterol (mg/dl)	51.8 ± 1.8	53.7 ± 3.4	49.9 ± 2.4	51.7 ± 3.4	0.693
LDL cholesterol (mg/dl)	119.2 ± 3.7	118.7 ± 7.1	126.9 ± 5.6	112.5 ± 6.1	0.276
TG (mg/dl)	166.5 ± 17.2	144.5 ± 14.0	164.8 ± 29.4	188.8 ± 39.4	0.581
Medications					
Metformin (%)	75.0	81.3	75.0	68.8	0.717
Thiazolidinedione (%)	2.1	0	6.3	0	0.360
Sulfonylurea (%)	33.3	25.0	37.5	37.5	0.687
DPP-4 inhibitor (%)	82.6	81.3	75.0	81.3	0.881
α-glucosidase inhibitor (%)	22.9	25.0	12.5	31.3	0.438
Insulin (%)	12.5	12.5	12.5	12.5	1.000
Lipid-lowering drugs (%)	58.3	68.8	37.5	68.8	0.117

The patients were devided into tertiles according to percentage of body weight change from baseline. Tertile 1 was defined as those with the highest tertile of body weight reduction, Tertile 2 as the intermediate, and Tertile 3 as the lowest. BMI, body mass index; SBP, systolic blood pressure; DBP, diastolic blood pressure; FPG, fasting plasma glucose; HbA_1c_, hemoglobin A_1c_; ALT, alanine aminotransferase; γ-GTP, γ-glutamyl transpeptidase; HDL, high density lipoprotein; LDL, low density lipoprotein; TG, triglyceride; DPP-4, dipeptidyl peptidase-4. Data are mean ± SEM. *p* values are calculated by analysis of variance (ANOVA) among three tertiles.

Systolic and diastolic blood pressure were decreased in Total (S4E and S4F Fig). Whereas LDL cholesterol was unchanged, HDL cholesterol showed an increasing trend and TG showed a decreasing trend in Total (S5C–S5E Fig).

## Discussion

In the present study, we demonstrated that ipragliflozin improved hepatic steatosis irrespective of body weight reduction in obese mice with insulin resistance. Ipragliflozin increased appetite and energy intake in the obese mice, which attenuated body weight reduction by urinary calorie loss. In patients with T2DM, ipragliflozin improved liver dysfunction irrespective of body weight reduction. These findings suggest a novel effect of SGLT2 inhibitors on lipid accumulation in the body, and a clinical implication for the therapeutic effect on hepatic steatosis in patients with T2DM. Based on the findings from the animal study, we consider that some of these effects resulted from their insulin-independent actions on lowering blood glucose.

Recently some papers reporting the effects of SGLT2 inhibitors on HFD-fed mice have been published. Treatment with an SGLT2 inhibitor tofogliflozin reduced HFD-induced body weight gain as well as hepatic steatosis, when it is administered for 20 weeks beginning at the start of HFD feeding [20]. In contrast, 4-week treatment of remogliflozin following 11-week of HFD feeding attenuated hepatic steatosis without changing body weight gain [21]. Although composition and starting age of HFD in the report were not identical, the protocol of remogliflozin treatment in the study resulted in similar findings to our study. Other recent report has shown that luseogliflozin decreased liver weight and serum ALT levels in STZ-treated mice fed a HFD, without affecting body weight gain [15]. Taken together, these observations suggest that the study protocol, especially in the timing of administration of SGLT2 inhibitors during HFD feeding and a mouse model, could affect body weight gain. In addition, it is possible that pharmacologic differences among SGLT2 inhibitors resulted in the variety of phenotypes. Further studies are necessary to ascertain the issue.

Ipragliflozin increased appetite and energy intake in HFD-fed mice, which attenuated body weight reduction by urinary calorie loss. It is independent of leptin action because ipragliflozin promoted hyperphagia and diminished the change of body weight in leptin-deficient *ob/ob* mice. Our observations are consistent with previous reports in mice [20, 21, 22] and rats [13] treated with SGLT2 inhibitors. Because the amount of increased energy intake induced by ipragliflozin treatment was roughly comparable to that of urinary calorie loss in our study, it is likely that ipragliflozin enhanced appetite without affecting other metabolic activities such as thermogenesis and locomotor activity. These observations imply that there are mechanisms in response to urinary glucose excretion to enhance appetite, and further studies are required to elucidate the detailed mechanisms.

Despite a lack of body weight reduction, ipragliflozin markedly improved hepatic steatosis in both HFD-fed and *ob/ob* mice. Lipid accumulation in the liver occurs due to an imbalance between hepatic TG synthesis and fatty acid oxidation. Both insulin and glucose stimulate gene expression of *Srebp1c*, a major transcription factor that positively regulates *de novo* lipogenic enzymes in the liver [23–25]. *De novo* lipogenesis is largely involved in inducing hepatic steatosis as demonstrated in a previous report showing that SREBP-1 ablation protected *ob/ob* mice from hepatic steatosis irrespective of adiposity and systemic glucose metabolism [26]. In the present study, ipragliflozin attenuated hyperinsulinemia probably via improving hyperglycemia in obese mice with insulin resistance, associated with reduced gene expression of *de novo* lipogenesis including *Srebp1c* and *Acc1* in the liver. In addition, elevation of fasting serum 3-hydroxybutyrate levels suggest enhanced fatty acid oxidation in the liver of ipragliflozin-treated mice. Although expression of β-oxidation-related genes were not affected by ipragliflozin treatment in the liver, it could not deny the possibility of increased fatty acid oxidation because CPT1 activity is allosterically regulated by malonyl-CoA synthesized via ACC [27]. Therefore, we consider that both decrease of *de novo* lipogenesis and increase of fatty acid oxidation contribute to improve hepatic steatosis by ipragliflozin treatment.

It has been demonstrated that hepatic steatosis is associated with the loss of epididymal fat mass in HFD-fed mice [28]. We and others have reported that fibrosis of white adipose tissue limits its lipid-storage capacity via inhibiting adipocyte hypertrophy, which has a role in ectopic lipid accumulation in the liver [18, 29]. In humans, lipodystrophy is known to be a similar condition characterized by the loss of adipose tissue with severe hepatic lipid accumulation [30]. Consistent with these observations, the present study has shown that ipragliflozin increased epididymal fat weight in HFD-fed mice, with an inverse correlation between liver and epididymal fat weights. It suggests that ipragliflozin treatment promoted normotopic fat accumulation in the epididymal fat and prevented ectopic fat accumulation in the liver.

In the epididymal fat of ipragliflozin-treated mice, increased adipocyte size and reduced number of CLS were observed. It suggests that ipragliflozin increased the lipid-storage capacity of adipocytes and inhibited adipocyte death followed by macrophage accumulation. Our results are consistent with a recent study using adipocyte-specific inducible phosphatase and tensin homologue (PTEN)-knockout mice, which exhibit enhanced insulin signaling in adipocytes. Although PTEN-knockout mice gained more weight and adipose tissue during HFD feeding, they showed enhanced insulin sensitivity, improved hepatic steatosis, and reduced adipose tissue inflammation [31]. Therefore, we consider that ipragliflozin treatment enhanced adipocyte insulin signaling via improving hyperinsulinemia, thereby resulting in increased lipid-storage capacity in adipocytes. Since it has been reported that hyperglycemia causes inflammation and insulin resistance in adipocyte with oxidative stress-dependent manner [32], reducing hyperglycemia by ipragliflozin could additionally contribute to improve insulin resistance and inflammation in adipocytes. Because ipragliflozin treatment caused an elevation in fasting serum NEFA levels in HFD-fed mice, ipragliflozin appeared to enhance lipolysis in adipose tissue. However, the possible increase of lipolysis has a small net effect on adiposity in HFD-induced obese mice; increased lipid-storage capacity in adipocytes by improvement of insulin sensitivity may exceed enhanced lipolysis, resulting in an increase in adiposity.

The present study showed that 4-week ipragliflozin treatment increased plasma glucagon levels in obese mice, which could induce the expression of the gluconeogenic gene *Pck1* in the liver. The finding is consistent with previous studies in which SGLT2 inhibitors increased both plasma glucagon and endogenous glucose production in subjects with T2DM [33, 34]. A recent report has demonstrated that SGLT2 is expressed in glucagon-secreting α-cells of the pancreatic islets and that dapagliflozin directly promotes glucagon secretion in human α-cells [35]. Increased plasma glucagon levels possibly due to the direct effect of SGLT2 inhibition on α-cells may offset the glucose-lowering effects of ipragliflozin. In patients with T2DM, glucose-induced suppression of glucagon is impaired, causing hyperglucagonemia and elevated hepatic glucose production [36]. The chronic effects of increased plasma glucagon levels by SGLT2 inhibition in T2DM await further studies.

Although the association with the changes of body weight remains unclear, it has been reported that the SGLT2 inhibitor canagliflozin improves liver dysfunction in patients with T2DM, assessed by serum ALT and γ-GTP levels [37]. In our human study, approximately 20% of patients treated with ipragliflozin did not achieve more than 1% of body weight reduction. Nevertheless, consistent with our animal study, we confirmed that liver dysfunction was improved by ipragliflozin even in patients without noticeable body weight reduction. In humans, a recent study has demonstrated that the SGLT2 inhibitor empagliflozin elicited an adaptive increase of energy intake in patients with T2DM, which caused substantially less weight loss than that expected from the energy dissipated because of glycosuria [12]. In addition, it has been demonstrated that body weight reaches a plateau rather than a sustainable decrease during SGLT2 inhibitors treatment in clinical studies [12, 38]. It is therefore conceivable that ipragliflozin treatment increased energy intake to attenuate body weight reduction in a patient population with a small reduction of body weight. The present clinical study has limitations; lipid accumulation in the liver and adipose tissue were not evaluated by imaging analysis such as ultrasonography or computed tomography. Further studies are required to clarify the changes of lipid accumulation both in the liver and adipose tissue of patients with T2DM during SGLT2 inhibitors treatment.

The reason for significant increase of serum HDL-cholesterol in tertile 2 is unclear. Given that baseline serum HDL-cholesterol levels and frequency of baseline medication of lipid-lowering drugs are the lowest in Tertile 2 among 3 tertiles, we speculate that the ability of SGLT2 inhibitors to increase serum HDL-cholesterol levels [37, 39, 40] clearly emerged in Tertile 2.

Unlike in mice, our clinical study suggests that the effects of SGLT2 inhibitors on human body weight vary across individuals. In humans, higher brain function such as thought and action more significantly contribute to feeding behavior than that in mice [41]. It is therefore conceivable that the complex of behavioral, environmental, and physiologic factors could modify the changes of body weight through feeding behavior in humans during treatment of SGLT2 inhibitors. In addition, since experimental mice are able to access food all the time under *ad libitum* feeding, it is likely that elicited appetite by SGLT2 inhibitors directly links to food intake, resulting in increased energy intake.

In conclusion, ipragliflozin improved hepatic steatosis in obese mice and liver dysfunction in patients with T2DM irrespective of body weight reduction. Although ipragliflozin increased energy intake and epididylmal fat mass to attenuate body weight reduction in the obese mice, systemic glucose intolerance and tissue insulin resistance were markedly improved. The present study could provide new insight into the effects of SGLT2 inhibitors on hepatic steatosis, and SGLT2 inhibitors can be potentially considered as an optional treatment of T2DM patients with hepatic steatosis.

## Supporting Information

S1 Fig Metabolic characterization and hepatic steatosis of *ob/ob* mice treated with or without ipragliflozin. Seven-week-old WT and *ob/ob* (Ob) mice were given with the vehicle or 10mg/kg of ipragliflozin for 4 weeks. (A) Blood glucose, (B) food intake, and (C) body weight during ipragliflozin treatment. Weights of the (D) liver and (E) epididymal fat after 4 weeks of ipragliflozin treatment. (F) Hematoxylin and eosin (HE) staining and (G) triglyceride (TG) content of the liver. Original magnification, × 200. Scale bars, 100 μm. # *p* < 0.05, ## *p* < 0.01 vs WT/Veh; * *p* < 0.05, ** *p* < 0.01 vs Ob/Veh. *n* = 5.
(PDF)

S2 Fig Metabolic characterization and hepatic steatosis of ipragliflozin-treated mice pair-fed a HFD. Eight-week-old WT mice were fed a HFD for 16 weeks. Mice were given with the vehicle or 10mg/kg of ipragliflozin during last 4 weeks. The ipragliflozin-treated mice pair-fed a HFD were given the average amount of food consumed by the vehicle-treated mice fed a HFD *ad libitum* on the previous day. (A) Blood glucose and (B) body weight during ipragliflozin treatment. Weights of the (C) liver and (D) epididymal fat after 4 weeks of ipragliflozin treatment. (E) Hematoxylin and eosin (HE) staining and (F) triglyceride (TG) content of the liver. Fasting (G) blood glucose, (H) serum insulin and (I) plasma glucagon levels. Original magnification, × 200. Scale bars, 100 μm. Veh, vehicle; Ipra, ipragliflozin; PF, pair-fed. * *p* < 0.05, ** *p* < 0.01 vs HFD/Veh. *n* = 6–7.
(PDF)

S3 Fig Effect of ipragliflozin on insulin signaling in the liver of ipragliflozin-treated mice pair-fed a HFD. Immunoblots of phosphorylated Akt (S473) in the liver of vehicle-treated mice fed a HFD *ad libitum* and ipragliflozin-treated mice pair-fed a HFD and after 16 h of fasting. Veh, vehicle; Ipra, ipragliflozin; PF, pair-fed.
(PDF)

S4 Fig Changes in body weight, waist circumference, and blood pressure in all 48 patients during 24 weeks of ipragliflozin treatment. Changes in body weight in (A) percentage and (B) absolute value, (C) BMI, (D) waist circumference, (E) systolic and (F) diastolic blood pressure in all 48 patients during 24 weeks of ipragliflozin treatment. * *p* < 0.05, ** *p* < 0.01 vs baseline.
(PDF)

S5 Fig Changes in blood test parameters in all 48 patients during 24 weeks of ipragliflozin treatment. Changes in (A) FPG, (B) HbA1c, serum levels of (C) HDL cholesterol, (D) LDL cholesterol, (E) TG, (F) ALT, and (G) γ-GTP in all 48 patients during 24 weeks of ipragliflozin treatment. * *p* < 0.05, ** *p* < 0.01 vs baseline.
(PDF)

S6 Fig Changes in body weight, blood pressure, lipids, and γ-GTP in Tertile 1–3 during 24 weeks of ipragliflozin treatment. Changes in (A) body weight, (B) systolic and (C) diastolic blood pressure, serum levels of (D) HDL cholesterol, (E) LDL cholesterol, (F) TG, and (G) γ-GTP in Tertile 1–3 during 24 weeks of ipragliflozin treatment. * *p* < 0.05, ** *p* < 0.01 vs baseline.
(PDF)

S7 Fig Changes in serum ALT levels, and correlation between changes in serum ALT levels and body weight after 24 weeks of ipragliflozin treatment in T2DM patients with hepatic steatosis. (A) Changes in serum ALT levels during 24 weeks of ipragliflozin treatment, and (B) correlation between changes in serum ALT levels and body weight after 24 weeks of ipragliflozin treatment in T2DM patients with hepatic steatosis. ** *p* < 0.01 vs baseline.
(PDF)

S1 Table List of primers. (PDF)

S2 Table Baseline characteristics of T2DM patients with hepatic steatosis. (PDF)
