# Supplementary figures and images for: Ipragliflozin Improves Hepatic Steatosis in Obese Mice and Liver Dysfunction in Type 2 Diabetic Patients Irrespective of Body Weight Reduction
Source: PLoS One. 2016 Mar 15;11(3):e0151511. doi: 10.1371/journal.pone.0151511 (PMC4792392; doi:10.1371/journal.pone.0151511)

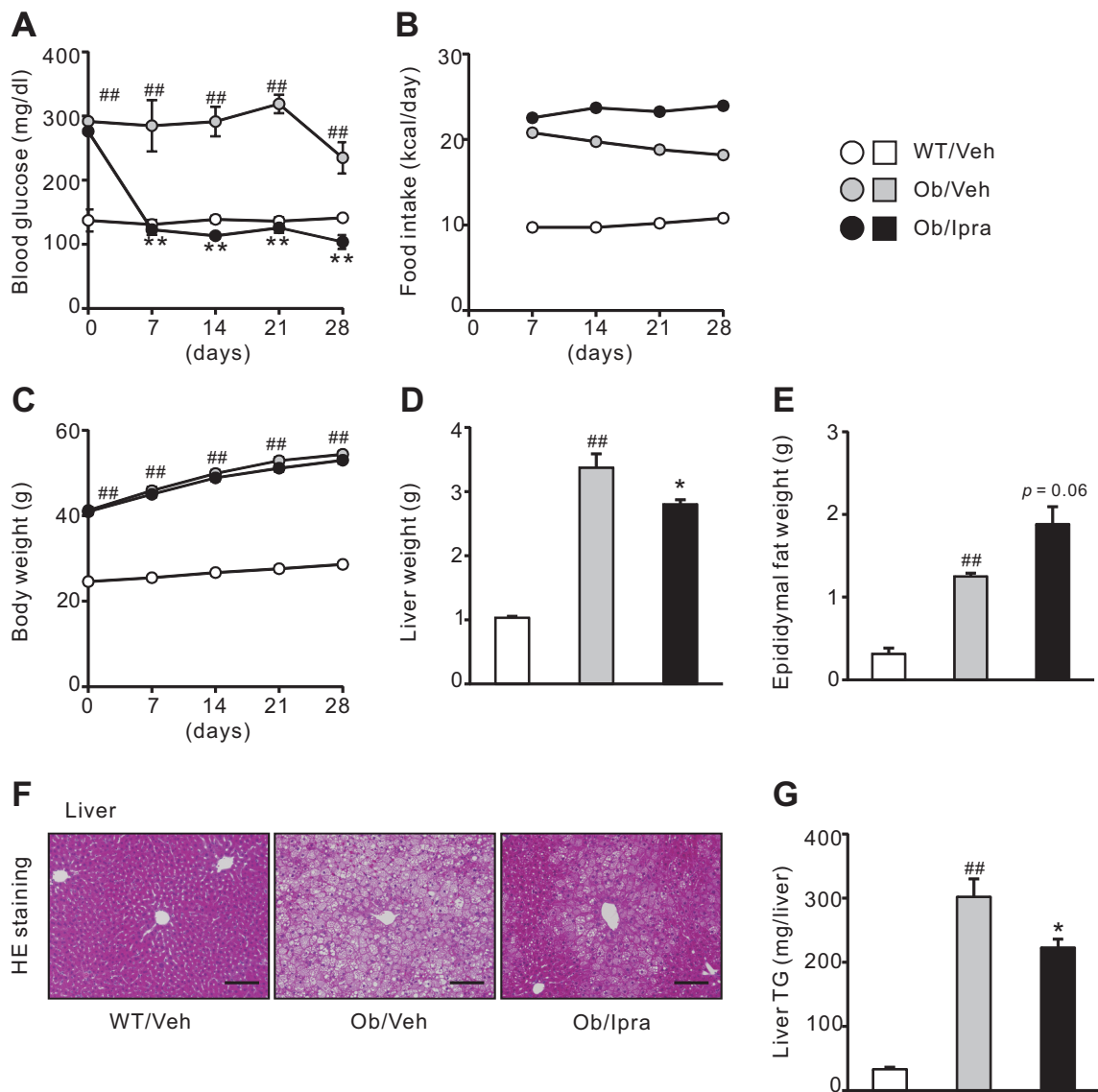

Supplemental Figure 1

Supplement: S1 Fig — Seven-week-old WT and ob/ob (Ob) mice were given with the vehicle or 10mg/kg of ipragliflozin for 4 weeks. (A) Blood glucose, (B) food intake, and (C) body weight during ipragliflozin treatment. Weights of the (D) liver and (E) epididymal fat after 4 weeks of ipragliflozin treatment. (F) Hematoxylin and eosin (HE) staining and (G) triglyceride (TG) content of the liver. Original magnification, × 200. Scale bars, 100 μm. # p < 0.05, ## p < 0.01 vs WT/Veh; * p < 0.05, ** p < 0.01 vs Ob/Veh. n = 5. (PDF) [file pone.0151511.s001.pdf]

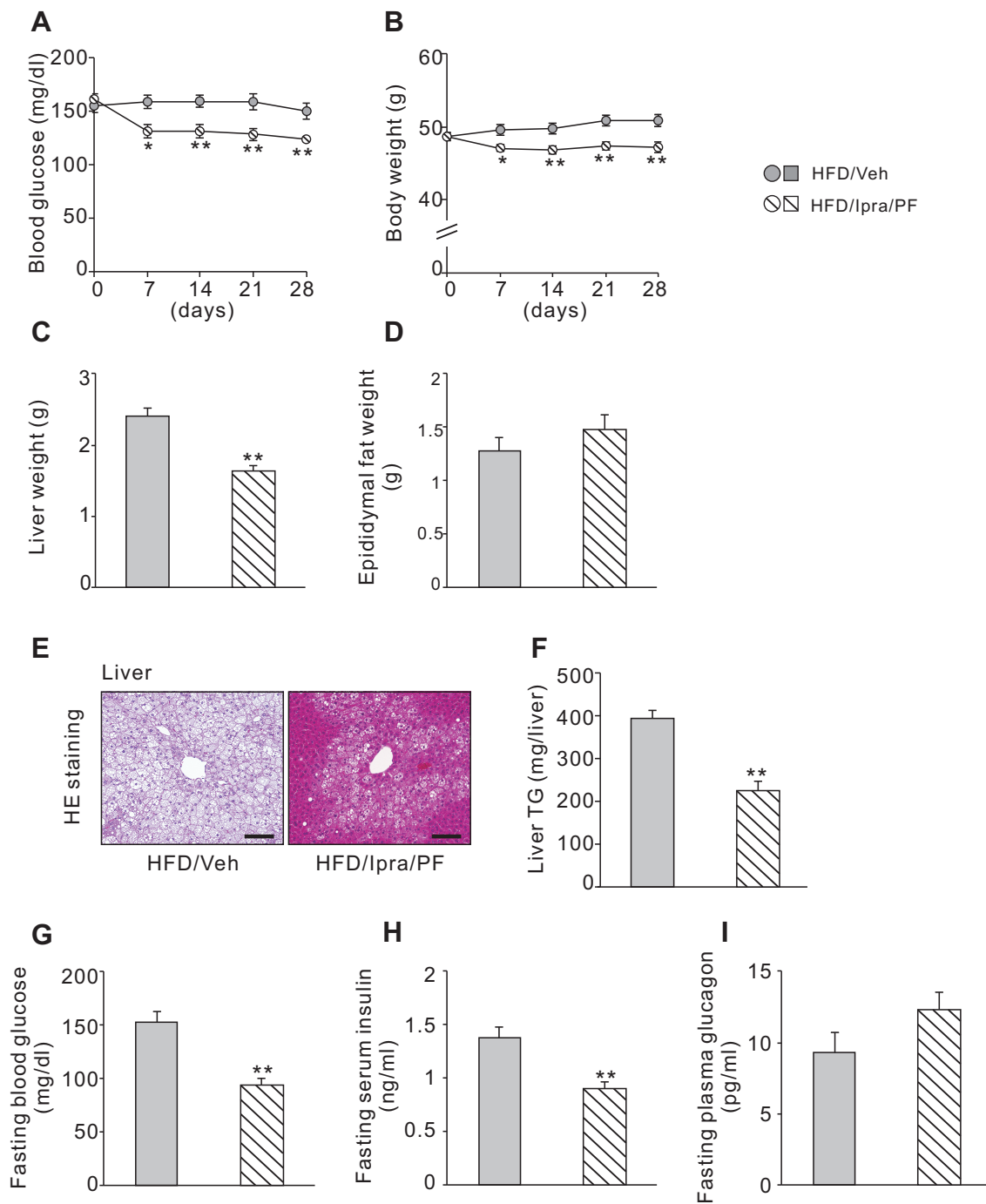

**Supplemental Figure 2**

Supplement: S2 Fig — Eight-week-old WT mice were fed a HFD for 16 weeks. Mice were given with the vehicle or 10mg/kg of ipragliflozin during last 4 weeks. The ipragliflozin-treated mice pair-fed a HFD were given the average amount of food consumed by the vehicle-treated mice fed a HFD ad libitum on the previous day. (A) Blood glucose and (B) body weight during ipragliflozin treatment. Weights of the (C) liver and (D) epididymal fat after 4 weeks of ipragliflozin treatment. (E) Hematoxylin and eosin (HE) staining and (F) triglyceride (TG) content of the liver. Fasting (G) blood glucose, (H) serum insulin and (I) plasma glucagon levels. Original magnification, × 200. Scale bars, 100 μm. Veh, vehicle; Ipra, ipragliflozin; PF, pair-fed. * p < 0.05, ** p < 0.01 vs HFD/Veh. n = 6–7. (PDF) [file pone.0151511.s002.pdf]

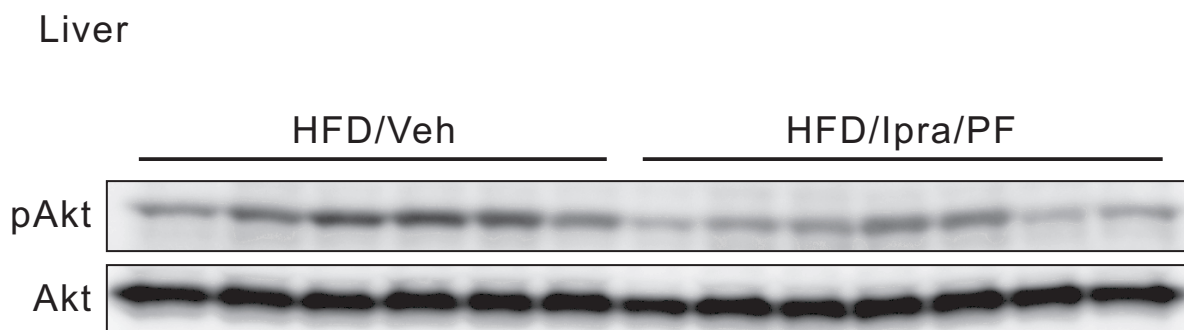

**Supplemental Figure 3**

Supplement: S3 Fig — Immunoblots of phosphorylated Akt (S473) in the liver of vehicle-treated mice fed a HFD ad libitum and ipragliflozin-treated mice pair-fed a HFD and after 16 h of fasting. Veh, vehicle; Ipra, ipragliflozin; PF, pair-fed. (PDF) [file pone.0151511.s003.pdf]

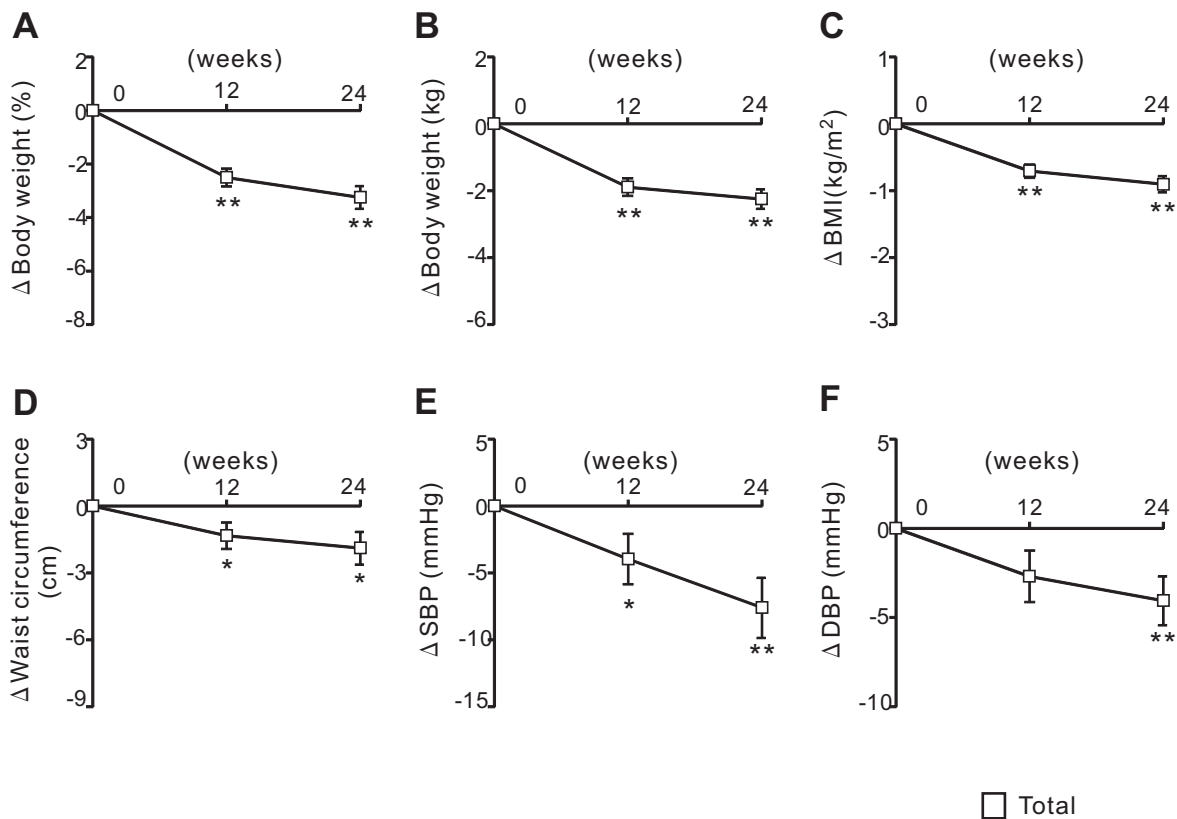

Supplemental Figure 4

Supplement: S4 Fig — Changes in body weight in (A) percentage and (B) absolute value, (C) BMI, (D) waist circumference, (E) systolic and (F) diastolic blood pressure in all 48 patients during 24 weeks of ipragliflozin treatment. * p < 0.05, ** p < 0.01 vs baseline. (PDF) [file pone.0151511.s004.pdf]

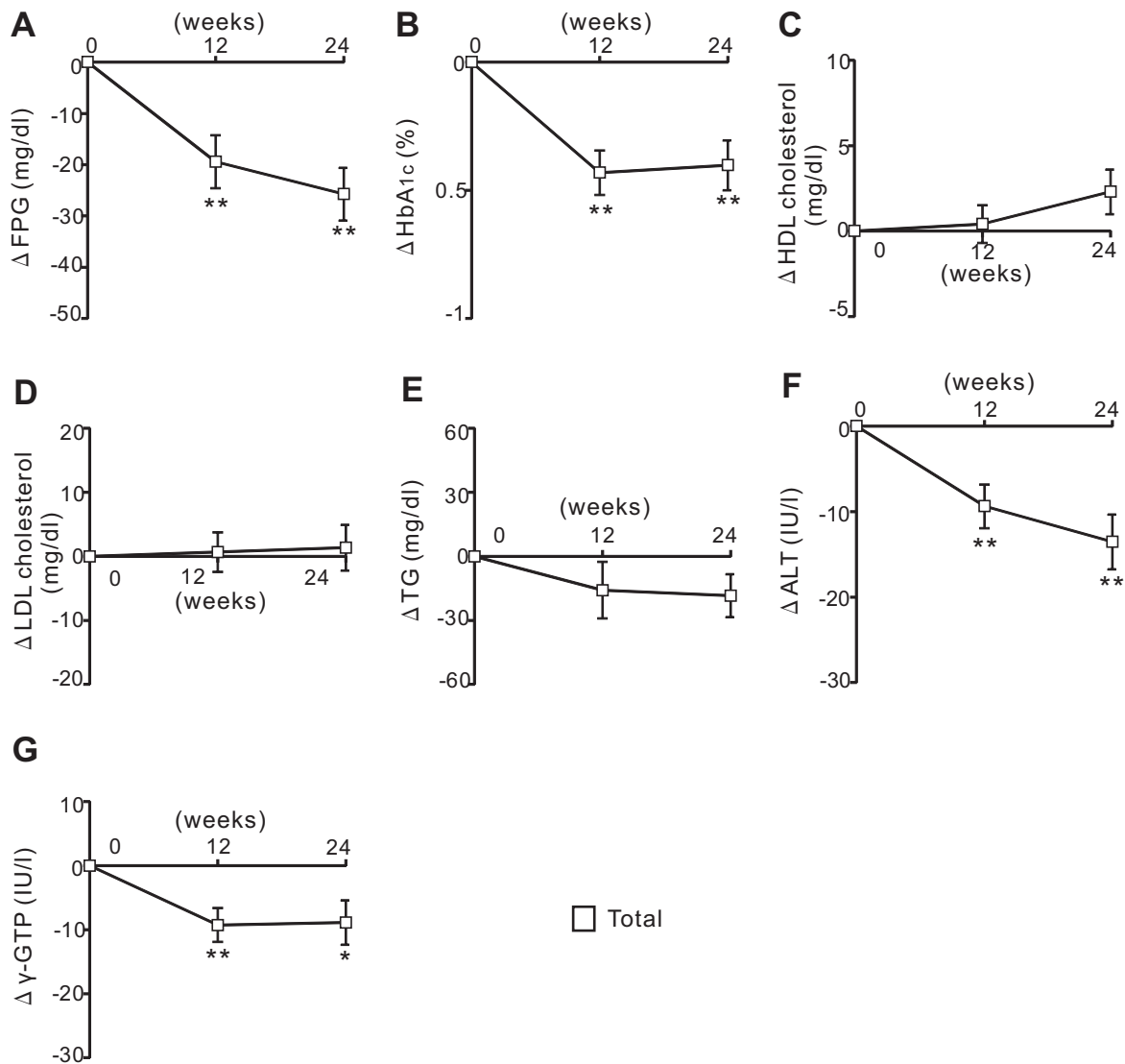

Supplemental Figure 5

Supplement: S5 Fig — Changes in (A) FPG, (B) HbA1c, serum levels of (C) HDL cholesterol, (D) LDL cholesterol, (E) TG, (F) ALT, and (G) γ-GTP in all 48 patients during 24 weeks of ipragliflozin treatment. * p < 0.05, ** p < 0.01 vs baseline. (PDF) [file pone.0151511.s005.pdf]

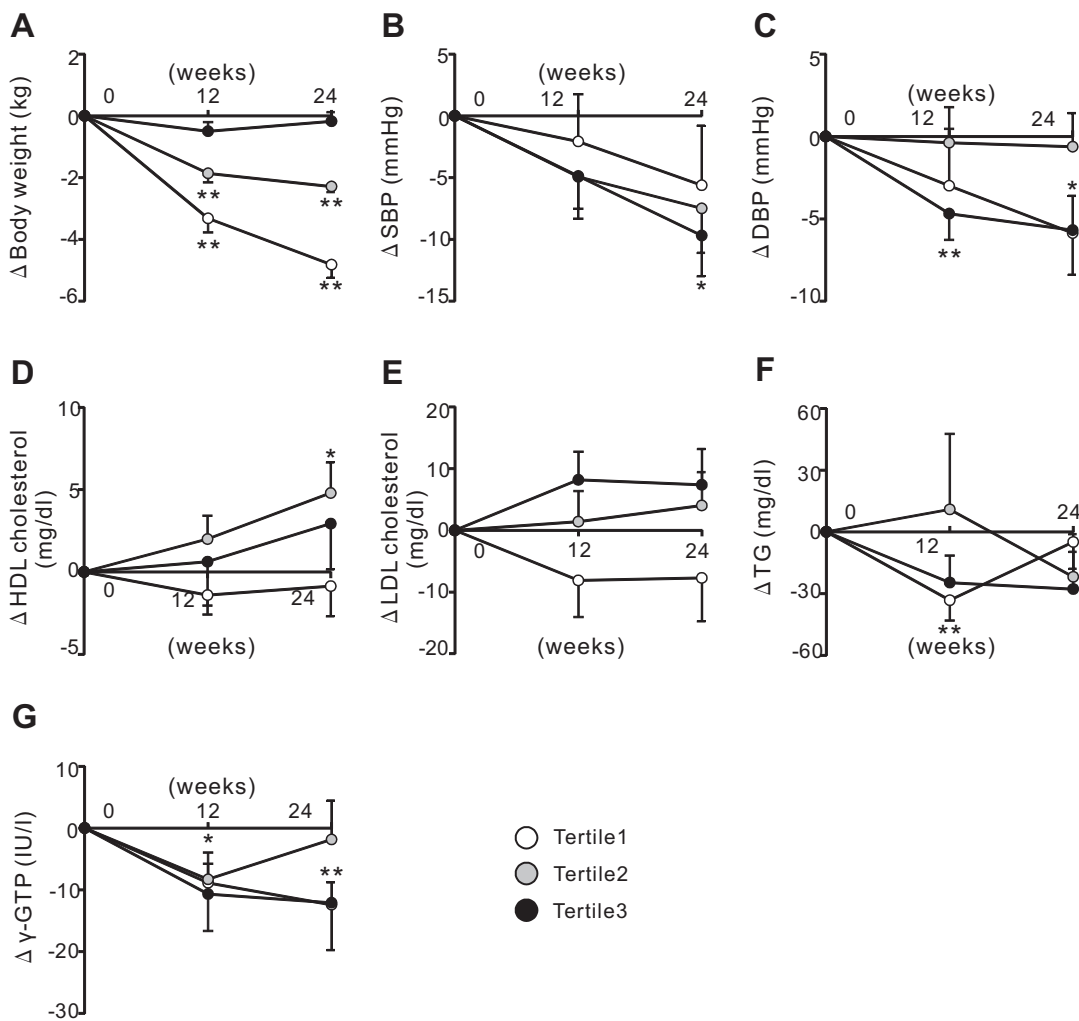

**Supplemental Figure 6**

Supplement: S6 Fig — Changes in (A) body weight, (B) systolic and (C) diastolic blood pressure, serum levels of (D) HDL cholesterol, (E) LDL cholesterol, (F) TG, and (G) γ-GTP in Tertile 1–3 during 24 weeks of ipragliflozin treatment. * p < 0.05, ** p < 0.01 vs baseline. (PDF) [file pone.0151511.s006.pdf]

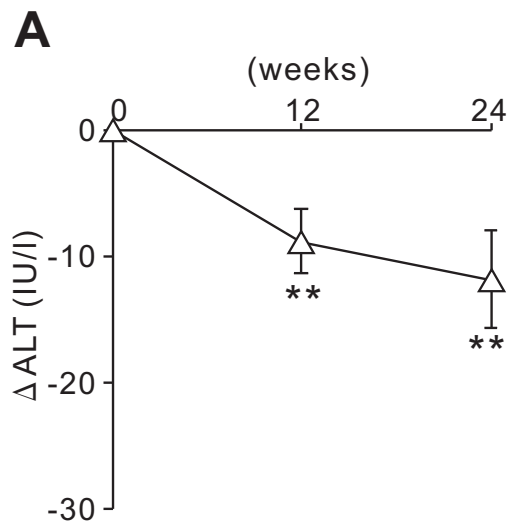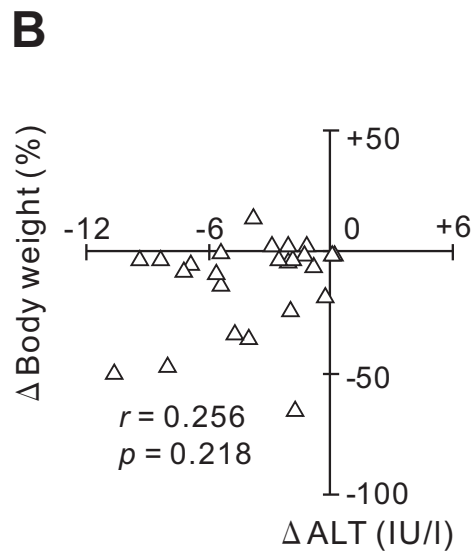

**Supplemental Figure 7**

Supplement: S7 Fig — (A) Changes in serum ALT levels during 24 weeks of ipragliflozin treatment, and (B) correlation between changes in serum ALT levels and body weight after 24 weeks of ipragliflozin treatment in T2DM patients with hepatic steatosis. ** p < 0.01 vs baseline. (PDF) [file pone.0151511.s007.pdf]
