# Supplementary material for: Ipragliflozin Improves Hepatic Steatosis in Obese Mice and Liver Dysfunction in Type 2 Diabetic Patients Irrespective of Body Weight Reduction
Source: PLoS One. 2016 Mar 15;11(3):e0151511. doi: 10.1371/journal.pone.0151511 (PMC4792392; doi:10.1371/journal.pone.0151511)
Supplement: S1 Table — (PDF) [file pone.0151511.s008.pdf]

S1 Table. List of primers

36b4	Fw	GGCCCTGCACTCTCGCTTTC
	Rv	TGCCAGGACGCGCTTGT
Srebp1c	Fw	CGGAAGCTGTCGGGGTAG
	Rv	GTTGTTGATGAGCTGGAGCA
Fasn	Fw	CCTGGATAGCATTCCGAACCT
	Rv	AGCACATCTCGAAGGCTACACA
Acc1	Fw	TGAGATTGGCATGGTAGCCTG
	Rv	CTCGGCCATCTGGATATTCAG
Scd1	Fw	ACGCCGACCCTCACAATTC
	Rv	CAGTTTTCCGCCCTTCTCTTT
Acox	Fw	GCCTTTGTTGTCCCTATCCGT
	Rv	CGATATCCCCAACAGTGATGC
Cpt1a	Fw	CCTGCATTCCTTCCCATTTG
	Rv	TGCCCATGTCCTTGTAATGTG
G6pc	Fw	CACCTGTGAGACCGGACCA
	Rv	GACCATAACATAGTATACACCTGCTGC
Pck1	Fw	CCACAGCTGCTGCAGAACA
	Rv	GAAGGGTCGCATGGCAAA
F4/80	Fw	CTTTGGCTATGGGCTTCCAGTC
	Rv	GCAAGGAGGACAGAGTTTATCGTG
Cd11c	Fw	GCCATTGAGGGCACAGAGA
	Rv	GAAGCCCTCCTGGGACATCT
Cd206	Fw	CGGTGAACCAAATAATTACCAAAAT
	Rv	GTGGAGCAGGTGTGGGCT

Tnf	Fw	ACCCTCACACTCAGATCATCTTC
	Rv	TGGTGGTTTGCTACGACGT
Il10	Fw	GCGCTGTCATCGATTTCTCC
	Rv	CACCTGCTCCACTGCCTTG
Adipoq	Fw	ATGGCAGAGATGGCACTCCT
	Rv	CCTTCAGCTCCTGTCATTCCA
