# Supplementary material for: Ipragliflozin Improves Hepatic Steatosis in Obese Mice and Liver Dysfunction in Type 2 Diabetic Patients Irrespective of Body Weight Reduction
Source: PLoS One. 2016 Mar 15;11(3):e0151511. doi: 10.1371/journal.pone.0151511 (PMC4792392; doi:10.1371/journal.pone.0151511)
Supplement: S2 Table — (PDF) [file pone.0151511.s009.pdf]

S2 Table. Baseline characteristics of T2DM patients with hepatic steatosis

n (Male/Female)	25 (11/14)
Age (year)	57.1 ± 2.6
Duration of diabetes (year)	8.8 ± 1.5
Body weight (kg)	74.7 ± 2.8
BMI (kg/m^2)	28.1 ± 0.9
Waist circumference (cm)	97.4 ± 1.9
SBP (mmHg)	133.9 ± 2.9
DBP (mmHg)	79.4 ± 1.6
FPG (mg/dl)	150.6 ± 9.4
HbA_{1c} (%)	7.62 ± 0.20
ALT (IU/l)	41.0 ± 5.7
γ-GTP (IU/l)	55.9 ± 9.5
HDL cholesterol (mg/dl)	50.4 ± 2.3
LDL cholesterol (mg/dl)	115.0 ± 4.7
TG (mg/dl)	159.4 ± 13.9
Medications	
Metformin (%)	76.0
Thiazolidinedione (%)	4.0
Sulfonylurea (%)	28.0
DPP-4 inhibitor (%)	84.0
α-glucosidase inhibitor (%)	20.0
Insulin (%)	12.0
Lipid-lowering drugs (%)	68.0

BMI, body mass index; SBP, systolic blood pressure; DBP, diastolic blood pressure; FPG, fasting plasma glucose; HbA_{1c}, hemoglobin A_{1c}; ALT, alanine aminotransferase; γ-GTP, γ-glutamyl transpeptidase; HDL,

high density lipoprotein; LDL, low density lipoprotein; TG, triglyceride; DPP-4, dipeptidyl peptidase-4.

Data are mean ± SEM.
